# The cost of cancer treatment in Portugal

**DOI:** 10.3332/ecancer.2017.765

**Published:** 2017-09-06

**Authors:** José Machado Lopes, Francisco Rocha Gonçalves, Marina Borges, Patrícia Redondo, José Laranja-Pontes

**Affiliations:** 1 Portuguese Institute of Oncology at Porto (IPO Porto), Rua Dr. António Bernardino de Almeida, 4200-072 Porto, Portugal; 2 ENSP-Universidade Nova de Lisboa, Av. Padre Cruz, 1600-560, Portugal.

**Keywords:** cancer, health, economics, costs, health technology assessment

## Abstract

**Introduction:**

Cancer is the second most common cause of death in Portugal, with 24.3% of these deaths caused by malignant neoplasms. The strong impact on lost productivity and rising treatment costs make cancer a priority. In order to understand, compare, and control costs by promoting transparency in the health system, it is vital to analyse the cost of oncological diseases. This study aims to estimate the economic burden associated with the treatment of cancer in Portugal by calculating the direct medical costs.

**Materials and methods:**

A prevalence-based study was conducted. The approaches used to estimate the costs were the top-down and gross costing techniques. In order to identify, quantify, and value all of the costs associated with the treatment of cancer, several sources of data were consulted to obtain the most up-to-date information on hospital care and a modified Delphi Panel was created to obtain data on primary health care.

**Results:**

The annual cost of cancer treatment in Portugal amounted to 867 million euros, representing 5.5% of the total expenditure for health and 84 euros *per capita*. The main component of this cost is antineoplastic drugs, which account for 31.5% of the total.

**Discussion and conclusion:**

By comparing the costs calculated in this study with those of the single Portuguese study conducted in 2009 and the European study carried out in 2013, we found that the annual cost for cancer treatment increased by about 300 million euros. An increase in incidence and the rising cost of drugs can explain this difference.

## Introduction

Knowing the cost of cancer treatment is crucial to the management of the health units and health system. In Portugal, the prevalence of oncological diseases is increasing, with 134,272 cases recorded in 2012 [1]. This rising trend is directly related to the ageing of the population, an increase in life expectancy and innovations in oncology, which have an impact on the global survival of patients. The number of new cases of cancer per year has also increased and the * European Cancer Observatory* has concluded that the age-standardised incidence rate in Portugal was 336.5 per 100,000 inhabitants in 2012, amounting to 49,174 new cases [1].

Cancer is the second most common cause of death in Portugal, after cardiovascular disease, with 24.3% of the deaths caused by malignant tumours [2]. According to data published by the Organisation for Economic Cooperation and Development (OECD), 26,220 people died of cancer in 2014, of which 59.7% were men [3]. Most of the deaths in that same year were caused by lung, colorectal, stomach, and prostate cancers [3].

The burden of oncological diseases is linked to their incidence, prevalence and the mortality and is often measured by the number of disability-adjusted life years (DALYs). Oncological diseases accounted for 527,651 DALYs in 2015 [4].

In addition to the demographic aspects, the strong impact on lost productivity and the rising costs of treatment make cancer a priority at the economic level. The increase in the number of new treatment options and the increase in their associated costs are leading decision-makers to question the public and private financing capacity of health systems [5]. The introduction of new treatments, however, has led to a gradual improvement in the quality of life of patients, their state of health and in disease control, either in terms of survival or productivity, contributing to a decrease in death rates through cure or the control of diseases considered fatal in the past [5].

It is vital to analyse the costs associated with oncological diseases, in order to understand, compare, and control costs and results, promoting transparency in the health system. The purpose of the present study can be defined by the following question: how much does it cost to diagnose and treat cancer patients in Portugal?

A study was conducted in 2009 [6] to estimate the cost of cancer treatment in Portugal and compare it with equivalent costs in Europe and in the United States of America. It estimated that 565 million euros were spent on direct costs of cancer treatment in Portugal, representing 3.91% of the total health expenditure. Regarding the burden of the disease, it was concluded that 15.3% of DALYs were associated with cancer [6]. This is an example of the scarcity of cost estimates related to the disease in Portugal, which requires an update and a triangulation from a methodological point of view.

Undoubtedly, both in Portugal and in other European countries, cancer continues to be a major cause of death, and the incidence of carcinomas has reached epidemic proportions this century. In 2009, the estimated economic cost of cancer in Europe, including direct and indirect costs, reached around 126 billion euros, with cancer-related healthcare costs at 102 euros per citizen [7]. The same study for Portugal estimated costs of 2048 million euros, of which 564 million euros are direct costs, 1216 million euros are productivity losses and 268 million euros are informal care costs [7]. The cost per citizen was 53 euros [7].

Through this article, we plan to complete the previous studies with more current data using a strict methodology, and estimate the economic burden associated with cancer treatment in Portugal by calculating direct costs with scheduled and unscheduled outpatient care, day hospital sessions for medical treatment, radiotherapy sessions, hospitalisation, and the use of drugs. The analysis will be broadened to the private sector, including some private expenses, so the results are not limited to the scope of public financing and are more in line with the perspective of Portuguese society as a whole.

## Materials and methods

### Portuguese health system

The Portuguese health system is composed of three concomitant systems: the National Health Service (Serviço Nacional de Saúde – SNS), special public and private healthcare insurance schemes for certain professions (health subsystems), and private healthcare insurers [8]. The SNS is the most commonly used system. It was created in 1979, is universal, and financed by taxes [8]. It is also a national, general, and free system [8]. Healthcare providers are mostly public, both in primary and in hospital care.

The Health Ministry is responsible for allocating funds to the SNS. The overall budget for the SNS is distributed by the various institutions based on the historical expenditure of health center groups (Agrupamento de Centros de Saúde – ACES), the capitation of local health units (Unidades Locais de Saúde – ULS), and annual payer contacts (Hospitals) [8]. More recently, payment methods have been introduced to cover overall costs for some pathologies.

The care model in the oncology area is composed of three highly specialised centers (the Portuguese Oncology Institutes), which cover the entire geographical area of Portugal and are supplemented by the provision of care in general hospitals.

### Appraising costs in health care

Calculating health costs is a challenge due to the complexity involved in providing care. The treatment of the patient involves different types of resources (e.g., staff, equipment, space, and materials) with different aspects and costs. These resources are used from the first contact of the patient with the institution and continue throughout the treatment in clinical actions and administrative processes [9].

Two approaches can be used to calculate costs: bottom-up and top-down [10]. In the bottom-up approach, the costs are estimated by identifying the resources used directly in the treatment of the disease, which allows for specific unit costs per person to be calculated [11]. In the top-down approach, the estimates are based on information regarding relevant costs from comprehensive sources, such as general hospital accounting, which allows for the average cost per patient to be calculated [11]. The cost components can be identified by using the techniques of microcosting*,* in which all relevant components are specified at the most detailed level possible, and/or gross costing*,* in which the components are specified at an aggregate level [11].

The choice of the most appropriate technique depends on several factors, such as the purpose of the study, the time frame for its completion, or the availability of data in the different information systems [10].

The economic appraisal provides for studies regarding the cost of the disease (Cost of Illness (CoI)) and its main objectives are [12]: to calculate the economic burden of the disease on society; to identify the main cost components and their impact on the overall cost; to describe the clinical management of the disease at a national level; and to explain the variability of costs. Using the categories of epidemiological data as criteria, these studies may be classified as [12]: based on incidence (include costs incurred from the date of diagnosis, or from a specific event, for all patients with certain clinical characteristics, up to the end of a predefined time period or until death [12]); or based on prevalence (include the costs associated with all living patients with a certain pathology over a given period of time [12]).

When studies include only medical care costs, they are usually designated as ‘treatment cost studies’ [13].

Regardless of the type of study, it will be important to identify and quantify at an early stage the resources needed for what is intended to be analysed [14].

In the assessment of health technologies, costs may be classified into three categories [15–17]: direct, indirect, and intangible costs. Direct costs arise from the use of health care, either through the health system (medical) or through the patient (non-medical), such as: staff costs, materials, consumables, costs of drugs, costs of complementary diagnostic resources and therapies (Meios Complementares de Diagnóstico e Terapêutica - MCDT), etc [18]. Indirect costs correspond to losses to the user/patient and their employer due to loss of work or leisure capacity as a result of early morbidity or death caused by the disease, for example: a decrease in productivity due to the after effects of the illness, work absences, early retirement. Intangible costs refer to changes in the quality of life and the effects of the disease itself or resulting from its treatment, such as pain and suffering [15–17]. The costs to be included in each study depend on the perspective adopted (e.g., Health Ministry, Society, etc.) [15–17].

Under the scope of the economic appraisal, several data sources were used to obtain information about costs in Portugal [13]: the administrative database, population and provider surveys, primary data collection, analytical accounting, and price list. Different sources can be used in the same study [14].

### Methods

In this study, the costs of cancer treatment were estimated based on the prevalence of the disease. Direct medical (clinical) costs were considered for this study based on its objectives and type. The top-down approach was used for the estimates and the cost components were identified using the gross-costing technique. The methodology used to obtain the costs was based on that used for the study *Economic burden of cancer across the European Union: a population-based cost analysis*, by Luengo-Fernandez *et al* [7].

All of the costs associated with the treatment of cancer in a hospital environment are identified, quantified, and assessed, dividing them into the following types of activity: treatment and complementary diagnostic exams prescribed through primary care; scheduled and unscheduled outpatient care, day hospital visits for medical treatment, radiotherapy, hospitalisations, and medications (chemotherapy/immunotherapy/hormone therapy) in a hospital environment.

For hospital care, various data sources were used to obtain the most up-to-date information. For primary health care data, a modified *Delphi* Panel was used.

The data are representative of the Portuguese population, since it is based on the volume of care provided to the population as a whole, including public and private providers. The exceptions to this rule are explained in the following paragraphs. For the calculations, the values directly related to oncological pathology were considered by line of activity.

### Data sources for hospital care

In order to assess the cost of all cancers, specific aggregate data on the use of healthcare were obtained from comprehensive national and international databases such as: *benchmarking* of the SNS of the Central Administration of the Health System (Administração Central do Sistema de Saúde - ACSS), the National Authority of Medicines and Health Products, I.P. (Autoridade Nacional do Medicamento e Produtos de Saúde – INFARMED), the National Statistics Institute (Instituto Nacional de Estatística – INE), the General Directorate of Health (Direção Geral de Saúde – DGS), the OECD, the Statistical Office of the European Union (EUROSTAT) and Quintiles IMS. The information about the most recent year was collected for use with the indicators deployed to measure costs, as shown in Table 1.

Sources that give information related to the public and private sector and national information are given priority. The source of the data is as follows:

- Cancer patients discharged: the DGS value (2014) for episodes with a primary diagnosis of cancer, which includes only SNS hospitals; the public data found did not include the private sector;
- Total cancer patients discharged: the number of discharged patients available on the Benchmarking website of the SNS of ACSS in 2014;
- Treatment: the INE value (2014) was considered, given that the values of the remaining sources do not include the private sector; but do include outpatient and emergency room consultations;
- Day hospital sessions: the only source available (DGS (2014)) was used and did not include the private sector;
- Radiotherapy sessions: the INE (2014) value was considered, given that the DGS value does not include the private sector;
- Expenses with drugs: the Quintiles IMS (2015) values are used, because the data published by INFARMED only includes the SNS hospitals.

In the second-stage methodology, which was essential for the results to be obtained, the quantities were valued.

Unit costs were obtained through the Benchmark Terms for Hospital Contractualisation of 2016 of the ACSS and Annual Payer Contracts (Contratos-Programa – CP) of 2016, given that the level of aggregation used for the quantities does not allow the Ordinance to be used with the prices assigned by the SNS, and that the prices provided under the scope of the CPs use the institutional costs as a benchmark. The Analytical Accounting available on the ACSS is not used because the data are from 2009 and the information is incomplete. The Diagnosis-Related Group (DRG) price includes all services rendered under the regime of hospitalisation, both in the infirmary and in the intensive care units, and includes surgical procedures, medical treatments, MCDT, drugs, and accommodation costs involved in the same episode [19].

Table 2 shows the assumptions used for the calculation of unit costs.

Lastly, the total cost is obtained by multiplying the quantities by the unit costs.

### Data sources for primary health care

Since no data are available for expenses with cancer in primary care, we thought it best to use the *Delphi* method to answer questions arising from the literature review for which no statistical information was found.

A group of experts, including General and Family Practitioners from the primary care units in the main areas of the country, met to fill out a short questionnaire on oncological disease which was divided into two groups: before diagnosis and after diagnosis.

With this method, we were able to estimate the number of consultations held and the supplementary diagnostic methods prescribed in primary health care, using the mean values. The estimates were valued at the prices of Ordinance no. 234 of 7 August 2015 and extrapolated to all cancer patients using the five-year incidence and prevalence data for 2012 of the *European Cancer Observatory* (ECO) of the *International Agency for Research on Cancer* (IARC) [1] and from *Globocan* [20].

The software used to analyze the data was Microsoft Excel.

The costs obtained represent one year of treatment under the prevalence aspect. No update rate was used since the time horizon is only one year.

Sensitivity analyses were performed on the parameters with the highest associated degree of uncertainty.

## Results

Table 3 shows that the annual cost of cancer treatment in Portugal amounted to 867 million euros, representing 5.5% of the total health expenditure and 84 euros *per capita*.

Table 4 shows a breakdown of the data, a summary of the data sources, and the year to which the information relates.

**Figure 1.**
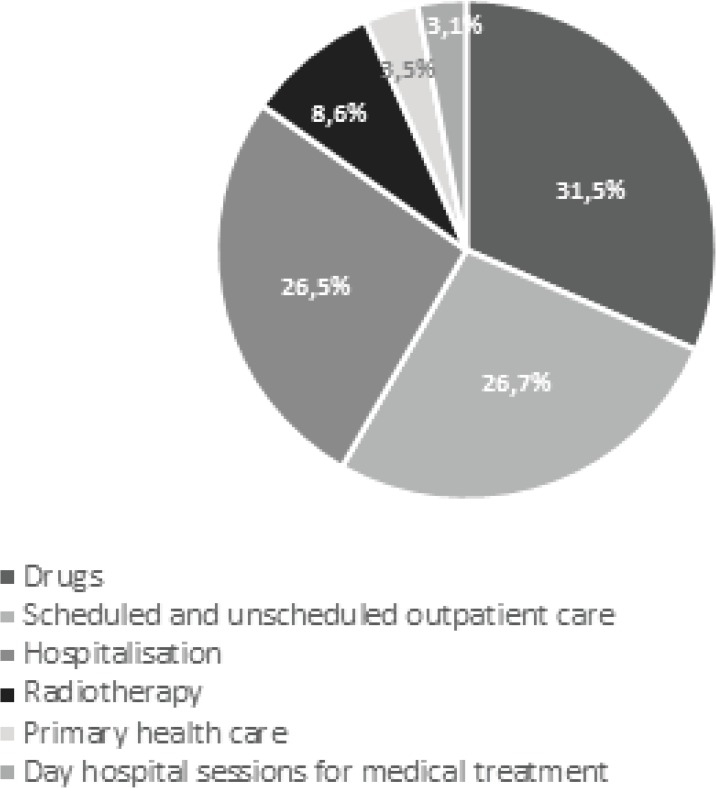
Percentage of annual costs for cancer treatment in Portugal, broken down by line of analysis.

Figure 1 shows the percentages of the values obtained, broken down into their various cost components. Drugs, hospitalisation, and outpatient care are the most significant cost components of cancer treatment, respectively, accounting for 31.5%, 26.7%, and 26.5% of the total. It should also be noted that Primary care represent only 3.5% of total costs.

### Sensitivity analysis

A sensitivity analysis was conducted to assess the effect of the variation on the parameters of the base model with a higher associated degree of uncertainty.

If we include in hospitalisation, the episodes that are associated with a primary and/or secondary diagnosis of cancer, that is, 122,047 (DGS (2014)), the total cost of cancer treatment shows a significant increase of 148,012,430 euros (17. 1%), reaching a total of 1015 million euros. This scenario was analysed because some of the episodes in which the oncological pathology appears only as secondary diagnosis are related to the provision of health care that is directly related to the treatment of this disease.

For the two lines of activity that do not include data on the private sector (day hospital sessions for medical treatment and hospitalisation), a second scenario was analysed, where variations of 6% in quantities were considered (a percentage identical to that recorded in the private sector for radiotherapy), with a total cost variation for cancer treatment of 15,433,654 euros (Table 5), that is, only 1.8%.

The number of outpatient visits is 3.5 times higher than that of the three Portuguese Oncology Institutes, so a sensitivity analysis was carried out on this line of activity. If we consider a hypothesis of a 20% reduction in the number of consultations, the total cost of treatment decreases by only 5.3% (46 million euros) to 821 million euros.

Lastly, given that the unit costs used are those of public hospitals, a sensitivity analysis was carried out in this field. A variation of between −10% and +10% in unit costs results in a respective variation of between −6% (−56 thousands euros) and +6% (+56 thousands euros) in the total cost of the treatment.

## Discussion

The methodology used allows us to identify, quantify, and value the main cost typologies (hospitalisation, consultations, and drugs) and their impact on total costs. It is intended for this estimate to translate into an added value for decision makers, so that cost policies can be defined for the areas with the greatest consumption of resources.

After comparing the results of this study with the values estimated in 2013 by Luengo-Fernandez *et al*. [7], which used a similar methodology, it is clear that the total costs obtained are higher by about 300 million euros, due to the following:

- there is a difference of 139 million in scheduled and unscheduled outpatient care resulting from the quantity estimates; the methodology used is similar, however, the data sources for discharged patients, for emergency cases, and for unit costs are different;
- there are two items under consideration, day hospital sessions for medical treatment and radiotherapy, which were not included in the aforementioned European study; the cost estimate associated with these lines of activity results in an increase in the total cost of about 100 million euros;
- there is a difference of 40 million euros in hospitalisation, due mostly to the fact that a higher unit cost was used in the current study, since we decided to use the value of patients discharged from the IPO, given that the complexity of cancer patients is higher than the national average; the European study used the base data of the analytical elements of the ACSS as a data source for the unit cost ;
- there is a difference of 26 million euros in the drugs line, since Luengo-Fernandez *et al*. used estimates based on data from Spain, while this study used data from Portugal.

The primary care line is the only one in which the European study estimate is higher than the one obtained in this study (about 13 million), which can be explained by differences in the methodology used. It should be noted that, in the first case, the estimates were obtained from the INE consultation data weighted by the number of cancer patients discharged in relation to the total number of patients discharged.

These differences resulted in an increase of 31 euros in the *per capita* cost (84 euros vs. 53 euros).

A comparison of the costs obtained in this study with those of article from Araújo’s *et al* [6], keeping in mind that methodological differences may compromise the comparison, shows an increase of about 300 million euros in the annual cost of cancer treatment.

With regard to the values obtained, it is worthwhile to point out that drugs are the main *driver* of costs. This is an item that merits special attention from decision-makers, since therapeutic innovations undoubtedly entail cost increases. As a line of future research, it will be important to calculate the impact of introducing new drugs into oncology in order to anticipate their effect for management purposes.

It should also be noted that primary care account for only 3.5% of total costs, because we are dealing with a pathology that is treated almost exclusively in a hospital environment. It is a chronic oncological pathology, with a growing prevalence and is treated more and more in outpatient setting, so it is necessary to consider the distribution of care between the two levels.

According to the sensitivity analysis, it is worth noting the impact on the cost of the treatment that results from only considering hospitalisation episodes in which the oncological pathology appears as a secondary diagnosis. This suggests the need for a future analysis aimed at identifying which of these episodes can be classified as directly related to cancer treatment.

The limitations encountered during the preparation of this study suggest that we reached a conservative estimate of the actual clinical cost of cancer in Portugal. The methodology used did not allow us to obtain data broken down by type of treatment: surgery, radiotherapy, chemotherapy, immunotherapy, or hormone therapy. The following lines of activity were not included: outpatient surgery, day hospital not related to antineoplastic therapy or radiotherapy sessions, stays in IPO home, home-based care, and dialysis. However, these are components with little impact on the total cost, if taking into account the experience of the IPO. The available data also did not allow us to obtain numbers for the public and private sectors on all lines (e.g., in hospitalisation and chemotherapy only public hospitals were considered). With regard to unit costs, the average costs of the three IPOs were extrapolated to the entire healthcare system. However, there is no literature that allows us to claim that the costs are similar. In addition, the consultation price was used as a *proxy* for the cost of a day hospital session, without group L medications (ATC code). According to the data available in the IPO Porto, this cost is not very different from the figures in the analytical accounts, which further justifies the option. Lastly, the price of the outpatient consultation includes part of the oral medication provided by the outpatient pharmacy.

## Conclusions

In conclusion, the annual cost of cancer treatment in Portugal is 867 million euros when only direct medical costs are considered. This represents a cost *per capita* of 84 euros, which is lower than the estimated 102 euros in the European Union. The main component of this cost is antineoplastic drugs, which account for a share of 31.5%.

Comparing the costs obtained in this study with those of Araújo’s *et al*.,s article [6] and those of the European study of Luengo-Fernandez [7], despite considering that the methodological differences may compromise the comparison, we found an increase of around 300 million euros in the annual cost of cancer treatment, which may be explained by an increase in incidence and the rising cost of drugs.

In the current context of the appearance of new molecules and other technologies with potential therapeutic benefits and higher prices, this study provides credible information on the treatment costs of various pathologies to decision-makers of health policies. This enables a more correct allocation of financial resources: for hospitals, knowing the cost of treatment components is an important management tool and a basis for prospective, clinical or economic studies, and for doctors, who in addition to the traditional obligation to seek what is ‘best for the patient,’ must also include a cost–benefit analysis of each new medication, thus contributing to the selection of truly differentiated and innovative technologies for patients and for society.

## Conflicts of interest

The authors declare that they have no conflict of interest regarding this article.

## Figures and Tables

**Table 1.** Analysis lines for direct cost appraisal.

Analysis lines	Indicators
Scheduled and unscheduled outpatient care	Cancer patients discharged/Total patients discharged × attendances
Day hospital sessions for medical treatment	Day hospital sessions
Radiotherapy	Radiotherapy sessions
Hospitalisation	Cancer patients discharged
Drugs	Drug expenses

**Table 2.** Assumptions for calculation of unit costs.

Lines of analysis	Assumptions
Scheduled and unscheduled outpatient care	Price of outside consultation for Group F (IPOs) (CP 2016);
Day hospital sessions for medical treatment	Price of outside consultations for Group F (IPOs) (CP 2016); the drugs administered in Group L (antineoplastic and immunomodulating agents), according to *the Anatomical Therapeutic Chemical Code* (ATC) of the World Health Organisation, are considered in the ‘drugs’ line;
Radiotherapy	Average price obtained by prices for simple and complex treatment (CP 2016), weighted by the quantities contracted for 2016 in all SNS hospitals with this line of activity;
Hospitalisation	Base price × CMI^*^ (CP 2016);
Drugs	Medication expenses (Quintiles IMS 2015).

* The Case Mix index (CMI) is the average of the CMI for the three IPOs in 2015 weighted by the number of patients discharged.

**Table 3.** Costs of cancer treatment in Portugal.

Population^*^	Total health expenditure (euros)^**^	Direct costs with cancer treatment (euros)	Direct costs per capita (euros)	Direct costs as % of health expenditure
10,341,330	15,887,738,000	867,000,731	84	5,5%

* INE data for 2015

** Health Satellite Account, INE, 2015

**Table 4.** Costs of cancer treatment in Portugal, broken down by line of analysis and data source.

Cost component	Quantity	Unit cost	Total value	Quantitative data source (year)	Price data source price (year)
Scheduled and unscheduled outpatient care	2,284,395	101.51 €	231,888,960 €	INE (2014)	CP (2016)
Day hospital sessions for medical treatment	268,768	101.51 €	27,282,640 €	DGS (2014)	CP (2016)
Radiotherapy	464,466	159.84 €	74,241,000 €	INE (2014)	CP (2016)
Hospitalisation	74,252	3,096.82 €	229,944,953 €	DGS (2014)	CP (2016) and ACSS (2015)
Drugs	n.a.	n.a.	273,446,918 €	IMS (2015)	IMS (2015)
Primary health care	n.a.	n.a.	30,196,259 €	Delphi Panel; IARC (2012) and Globocan (2012)	Ordinance 234/2015
**Total**	**—**	**—**	**867,000,731 €**	**—**	**—**

**Table 5.** Sensitivity analysis scenario 2.

Cost component	Base scenario	Variation (6%)
Day hospital sessions for medical treatment	27,282,640 €	1,636,958 €
Hospitalisation	229,944,953 €	13,796,697 €
**Total cost including all items**	**867,000,731 €**	**15,433,654 €**
